# Diagnosis and Management of Secondary HLH/MAS Following HSCT and CAR-T Cell Therapy in Adults; A Review of the Literature and a Survey of Practice Within EBMT Centres on Behalf of the Autoimmune Diseases Working Party (ADWP) and Transplant Complications Working Party (TCWP)

**DOI:** 10.3389/fimmu.2020.00524

**Published:** 2020-03-31

**Authors:** Robert David Sandler, Rachel Scarlett Tattersall, Helene Schoemans, Raffaella Greco, Manuela Badoglio, Myriam Labopin, Tobias Alexander, Kirill Kirgizov, Montserrat Rovira, Muhammad Saif, Riccardo Saccardi, Julio Delgado, Zinaida Peric, Christian Koenecke, Olaf Penack, Grzegorz Basak, John Andrew Snowden

**Affiliations:** ^1^Department of Rheumatology, Sheffield Teaching Hospitals NHS Foundation Trust, Sheffield, United Kingdom; ^2^Department of Hematology, University Hospitals Leuven, KU Leuven, Leuven, Belgium; ^3^Haematology and BMT Unit, San Raffaele Hospital (IRCCS), Milan, Italy; ^4^EBMT Paris Study Office, Department of Haematology, Hôpital Saint-Antoine, Paris, France; ^5^Department of Rheumatology and Clinical Immunology, Charité—Universitätsmedizin Berlin, Berlin, Germany; ^6^Russian Cancer Research Center NN Blokhin, Moscow, Russia; ^7^BMT Unit, Department of Hematology, August Pi i Sunyer Biomedical Research Institute (IDIBAPS), Barcelona, Spain; ^8^Manchester Royal Infirmary, Manchester, United Kingdom; ^9^Cell Therapy and Transfusion Medicine Unit, Careggi Hospital, Florence, Italy; ^10^School of Medicine, University of Zagreb, Zagreb, Croatia; ^11^University Hospital Centre Zagreb, Zagreb, Croatia; ^12^Department of Hematology, Hemostasis, Oncology and Stem Cell Transplantation, Hannover Medical School, Hanover, Germany; ^13^Department of Hematology, Oncology and Tumorimmunology, Charité—Universitätsmedizin Berlin, Berlin, Germany; ^14^Department of Hematology, Oncology and Internal Medicine, University Clinical Center of the Medical University of Warsaw, Warsaw, Poland; ^15^Department of Haematology, Sheffield Teaching Hospitals NHS Foundation Trust, Sheffield, United Kingdom

**Keywords:** GVHD, CAR-T cell, HSCT, HLH hemophagocytic lymphohistiocytosis, macrophage activation syndrome (MAS), ferritin, biomarkers

## Abstract

**Introduction:** Secondary haemophagocytic lymphohistiocytosis (sHLH) or Macrophage Activation Syndrome (MAS) is a life-threatening hyperinflammatory syndrome that can occur in patients with severe infections, malignancy or autoimmune diseases. It is also a rare complication of haematopoetic stem cell transplantation (HSCT), with a high mortality. It may be associated with graft vs. host disease in the allogeneic HSCT setting. It is also reported following CAR-T cell therapy, but differentiation from cytokine release syndrome (CRS) is challenging. Here, we summarise the literature and present results of a survey of current awareness and practice in EBMT-affiliated centres of sHLH/MAS following HSCT and CAR-T cell therapy.

**Methods:** An online questionnaire was sent to the principal investigators of all EBMT member transplant centres treating adult patients (18 years and over) inviting them to provide information regarding: number of cases of sHLH/MAS seen in their centre over 3 years (2016–2018 inclusive); screening strategies and use of existing diagnostic/classification criteria and treatment protocols.

**Results:** 114/472 centres from 24 different countries responded (24%). We report estimated rates of sHLH/MAS of 1.09% (95% CI = 0.89–1.30) following allogeneic HSCT, 0.15% (95% CI = 0.09–5.89) following autologous HSCT and 3.48% (95% CI = 0.95–6.01) following CAR-T cell therapy. A majority of centres (70%) did not use a standard screening protocol. Serum ferritin was the most commonly used screening marker at 78% of centres, followed by soluble IL-2 receptor (24%), triglycerides (15%), and fibrinogen (11%). There was significant variation in definition of “clinically significant” serum ferritin levels ranging from 500 to 10,000 μg/mL. The most commonly used criteria to support diagnosis were HLH-2004 (43%) and the H score (15%). Eighty percent of responders reported using no standard management protocol, but reported using combinations of corticosteroids, chemotherapeutic agents, cytokine blockade, and monoclonal antibodies.

**Conclusions:** There is a remarkable lack of consistency between EBMT centres in the approach to screening, diagnosis and management. Further research in this field is needed to raise awareness of and inform harmonised, evidence-based approaches to the recognition and treatment of sHLH/MAS following HSCT/CAR-T cell therapy.

## Background Review: sHLH/MAS in Relation to HSCT and CAR-T Cell Therapy

Secondary haemophagocytic lymphohistiocytosis (sHLH) is a life-threatening syndrome, seen in the context of haematological malignancy, infection, and autoimmunity/immune dysregulation (1). Secondary HLH is termed macrophage activation syndrome (MAS) when associated with rheumatological disease, typically in the context of systemic juvenile idiopathic arthritis (sJIA), adult onset Still's disease (AOSD), and systemic lupus erythematosus (SLE).

Secondary HLH is reported after both allogeneic and autologous haematopoetic stem cell transplantation (HSCT), particularly in association with graft vs. host disease (GVHD) in patients undergoing allogeneic HSCT (2–7). Infections, in particular Epstein-Barr virus (EBV) and cytomegalovirus (CMV) can be important triggers and mortality in all cases is high (2, 4, 5, 8). Only a few studies to date have addressed incidence of sHLH/MAS post HSCT, estimating ~3–4% (2, 6, 8). Once diagnosed, the mortality of sHLH/MAS in the allogeneic HSCT setting appears to be high, with rates of up 80% reported in recent studies (6, 7). Even though reports of sHLH/MAS following autologous HSCT appear to be rare, reports of death due to sHLH/MAS in patients with refractory JIA undergoing autologous HSCT have prompted changes in immunosuppressive and infectious prophylactic regimens, leading to decreased mortality (9).

Diagnosis of sHLH/MAS post-HSCT requires a high index of clinical suspicion in identifying hyperinflammation, particularly as features overlap those of severe sepsis or GVHD. Typically these include fever, several-lineage cytopenia, and multi-organ failure. Persistent fever in patients without an identified infective cause, or worsening fever in patients who have been treated for infection, should prompt consideration of sHLH/MAS (10).

Serum ferritin is a useful, readily available biomarker of sHLH/MAS and can be used to gauge response to treatment (2, 5, 11, 12). It is closely related to disease activity, and both maximum levels during sHLH/MAS, and a fall of less than 50% after treatment are associated with higher mortality (13–15). A retrospective paediatric study found serum ferritin levels of >10,000 μg/mL 90% sensitive and 96% specific for HLH, but its utility in the adult post-HSCT setting has not been validated (16). Serum ferritin > 10,000 μg/mL has been associated with poor survival in patients with GVHD, but this study did not investigate if these patients had sHLH/MAS (17). There is evidence that ferritin levels are not strongly associated with presence of GVHD, so may prove a useful biomarker allowing differentiation from sHLH/MAS (18, 19). Serum levels of soluble interleukin-2 (IL-2) receptor (sIL-2r) have emerged as an alternative diagnostic measure in adult patients with non-HSCT related sHLH/MAS but are not been validated in the post-HSCT setting (20). Furthermore, recent work has identified elevated serum levels of multiple cytokines and chemokines at the onset of sHLH/MAS following allogeneic HSCT, which may indicate a state of allo-reactivity, as seen in GVHD, which may precipitate sHLH/MAS (5). Histological identification of haemophagocytosis is recognised as a late feature and does not correlate as well as fever or serum ferritin with clinical diagnosis (21–23). Therefore demonstration of haemophagocytosis is not considered essential for diagnosis, and may only be detected if bone marrow samples are taken in the later stages of disease.

Various classification criteria exist for sHLH/MAS, some derived from familial HLH and others from rheumatological practice in JIA (summarised in Table 1) (24, 26, 27). A diagnostic calculator, the “H score,” takes into account clinical and laboratory features to calculate a percentage probability of sHLH/MAS in adults (25). With the lack of validated diagnostic criteria for sHLH/MAS in adult patients in general, and post-HSCT patients in particular, it is possible to take a pragmatic approach, utilising the “H score” whilst recognising its limitations. The H-score was based on a single-centre retrospective study of sHLH/MAS and of the 43% of included patients who had diagnosed haematological malignancy, it is not reported if any had already undergone HSCT. Studies of the performance of the H-score in detecting sHLH/MAS have been encouraging, particularly in the early clinical stages of the disease, where the H-score appears to outperform HLH-2004 criteria (28, 29).

**Table 1:** Use of published criteria to support the diagnosis of sHLH/MAS post-HSCT or CAR-T cell therapy.

**Published criteria**	**Components of criteria**	**Centres (%)**
HLH-2004 (for fHLH) (24)	Molecular diagnosis consistent with HLH or 5/8 of the following: Fever, splenomegaly, bi or tri-lineage cytopenia, hypertriglyceridaemia ± hypofibrinogenaemia, haemophagocytosis on bone marrow biopsy, no diagnosis of malignancy, low/absent NK cell activity, raised ferritin, raised sIL-2r	43
H-score (for all sHLH/MAS) (25)	Known underlying immunosuppression, fever, organomegaly, mono-, bi-, or tri-lineage cytopenia, ferritin, triglycerides, fibrinogen, AST, haemophagocytosis on bone marrow biopsy. Overall score predicts likelihood of sHLH/MAS	16
Takagi et al. (for SHLH/MAS post-HSCT)	2 major or 1 major and all 4 minor criteria required. Major criteria: (A) engraftment delay, primary or secondary failure or (B) histopathological evidence of haemophagocytosis. Minor criteria: fever, hepatosplenomegaly, elevated ferritin, elevated LDH.	10
PRINTO (for sHLH/MAS in sJIA)	Ferritin > 684 μg/L and 2 of: platelets <181 × 109, AST >48 U/L, triglycerides >256 mg/dL, fibrinogen <360mg/dL	1
MD Anderson (for sHLH/MAS post-CAR-T cell therapy)	Ferritin of > 10,000 μg/L and 2 of: grade > 3 increase in serum transaminases or bilirubin; grade > 3 oliguria or increase in serum creatinine; grade > 3 pulmonary oedema; or histological evidence of haemophagocytosis in bone marrow or organs	7
Combination of the above		23

Where post-HSCT patients are unwell, febrile, with a serum ferritin of >10 000 μg/L and present with no proven infection except for the presence of recognised triggers of HLH such as EBV and other herpes viral reactivations/infections, they can be considered in a “hyperinflammatory state” and should be considered for aggressive immunosuppression, as per published recommendations (1, 16, 30). Indicators of a poor prognosis include neurological dysfunction, acute kidney injury and acute respiratory distress (1).

Effective treatment of sHLH/MAS requires aggressive immunosuppression, controlling the hyperinflammatory state, in combination with targeted treatment addressing triggering factors. Prompt recognition and treatment is important and reduces mortality in cases of sHLH/MAS secondary to autoimmune disease (31).

Corticosteroids remain the cornerstone of induction treatment, although over half of patients may be steroid-resistant (32). Dramatic responses are reported with the addition of CSA in doses of 2–7 mg/kg/day (33, 34). Anakinra, an IL-1 antagonist, is effective in refractory sHLH/MAS and relatively safe in patients with sepsis (35, 36). Anakinra is now at the forefront of treatment in sJIA-triggered sHLH/MAS and shows promise in adult sHLH/MAS in the intensive care setting (37, 38). Intravenous immunoglobulin (IVIG) infusions may also be effective in steroid-resistant and EBV-triggered sHLH/MAS (39). Rituximab improves overall clinical outcomes and is an important part of EBV clearance in patients with EBV-triggered sHLH/MAS or EBV-driven malignancies (40, 41). Case reports of refractory sHLH/MAS, in patients who had not already undergone HSCT or CAR-T cell therapy, note complete responses with rabbit anti-thymocyte globulin (ATG) or DEP regimen (doxorubicin, etoposide, methylprednisolone) and partial responses with alemtuzumab (42).

A treatment protocol for sHLH/MAS accepting the heterogeneity of this syndrome and irrespective of preceding HSCT or CAR-T cell therapy has been recently published (1). First line treatment is with intravenous methylprednisolone (IVMP) 1g/day for 3–5 days plus IVIG 1g/kg for 2 days, which can be repeated at day 14. If there is evidence of established sHLH/MAS or clinical deterioration, Anakinra is added, 1–2 mg/kg daily increasing up to 8 mg/kg/day until sufficient clinical response. CSA is considered for early or in steroid-resistant disease. Etoposide should be considered in refractory cases. There should be parallel consideration of identifying and eradicating triggers, such as EBV, bacterial infection, and underlying malignancy, particularly lymphoma. There are no validated guidelines for treating sHLH/MAS post-HSCT and there are concerns about using the HLH-2004 protocol, especially with the inclusion of etoposide (43).

CAR-T cell therapy, whilst emerging as an effective treatment for both haematological and non-haematological malignancy, is associated with cytokine release syndrome (CRS), an acute toxicity resulting in hyperinflammation. Patients can present with CRS across a spectrum of severity, from low-grade constitutional symptoms to higher-grade systemic illness with multi-organ dysfunction and, in its most severe form, this can progress to fulminant sHLH/MAS. Neelapu et al. have proposed diagnostic criteria for sHLH/MAS in patients with CRS post-CAR-T cell therapy demonstrating peak serum ferritin measurement of >10,000 μg/L and two of the following findings: grade > 3 increase in serum transaminases or bilirubin; grade > 3 oliguria or increase in serum creatinine; grade > 3 pulmonary oedema or histological evidence of haemophagocytosis in bone marrow or organs (44). They also recommend specific treatment with corticosteroids and anti-IL-6 therapy (Tocilizumab or Siltuximab) alongside supportive care (44).

Against this background, we surveyed members of the European Society for Blood and Marrow Transplantation (EBMT) to:

1. Estimate the rates of sHLH/MAS recognised in their patients following HSCT or CAR-T cell therapy,
2. Review the classification criteria and screening methods used to identify sHLH/MAS and
3. Describe approaches to managing sHLH/MAS in these patients.

## Methods

A limited questionnaire with single and multiple-choice questions was distributed, in the form of web based survey (Eval&Go, Montpellier, France) to the principal investigators of all EBMT member centres treating adult patients aged 18 and over, with autologous or allogeneic HSCT and/or CAR-T cell therapy, for any indication. They were invited to complete the survey and provide information on the following aspects of sHLH/MAS post-HSCT or CAR-T cell therapy to reflect their centre's experience: number of cases of sHLH/MAS seen in their centre over 3 years (2016–2018 inclusive); screening strategies; use of existing diagnostic/classification criteria and treatment protocols (Appendix 1 in Supplementary Material).

Principal Investigators at all 472 EBMT member centres performing HSCT and/or CAR-T cell therapy in patients 18 years and above were invited for participation. All non-responders received a maximum of three e-mail reminders over a period of 3 months.

Quality checks were performed to avoid duplicate responses. Descriptive statistics were used as appropriate. Continuous data were summarised using descriptive statistics comprising of the number of subjects with data to be summarised (n), median, inter-quartile range (IQR), minimum (min), and maximum (max). Categorical variables were presented using counts and percentages.

We estimated the rates of sHLH/MAS by the ratio between the number of reported cases of sHLH/MAS and the number of HSCT procedures performed during the three-year period (2016–18) in the 114 returning centres (the denominator being derived from the EBMT registry, where there is mandatory reporting of all HSCT procedures according to full EBMT membership). For CAR-T cell therapy, individual centres provided the total number of procedures performed for use as the denominator.

## Results

A total of 114 centres from 24 countries returned the survey.

One twenty-nine cases (109 following allogeneic HSCT and 20 following autologous HSCT) of sHLH/MAS were reported by 114 centres which had performed 23 097 HSCT (9 972 allogeneic and 13 125 autologous). This corresponded to an estimated sHLH/MAS rate of 1.09% (CI 0.89–1.30%) and 0.15% (CI 0.09–5.89), after allogeneic and autologous HSCT, respectively. Seven cases of sHLH/MAS were reported in 201 patients having received CAR-T cell therapy, giving an estimated rate of 3.48% (CI 0.95–6.01).

A total of 108 responders completed the remainder of the survey and their responses were involved in further analysis.

## Screening for sHLH/MAS Following HSCT/CAR-T Cell Therapy

### Use of a Standard Screening Approach Following HSCT

One hundred and six centres responded to the questions, with 74 (70%) reporting using no agreed approach to screening for sHLH/MAS in their centre.

**Figure 1:**
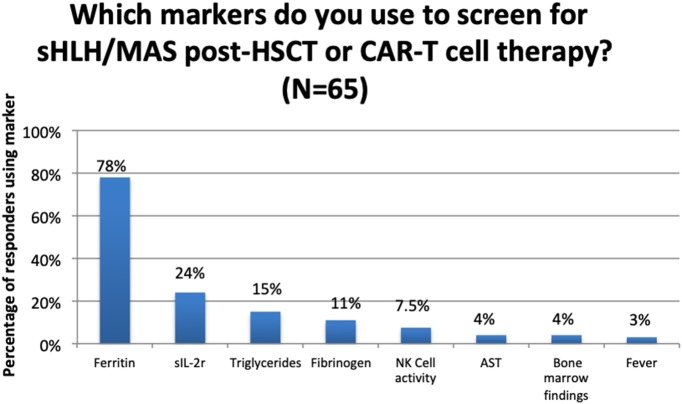
Use of clinical/laboratory markers to screen for sHLH/MAS post-HSCT or CAR-T Cell therapy.

Whilst only 32 centres reported using a standard protocol, 80 centres reported use of screening markers, with ferritin being the most reported biomarker in the multiple-choice options (Figure 1).

### Use of a Standard Screening Approach Following CAR-T Cell Therapy

For the 22 centres that performed CAR-T cell therapy, 4 (19%) reported no screening and 11 (52%) reported screening when there is clinical suspicion. Six centres (29%) reported unique routine screening protocols and one centre did not respond.

**Figure 2:**
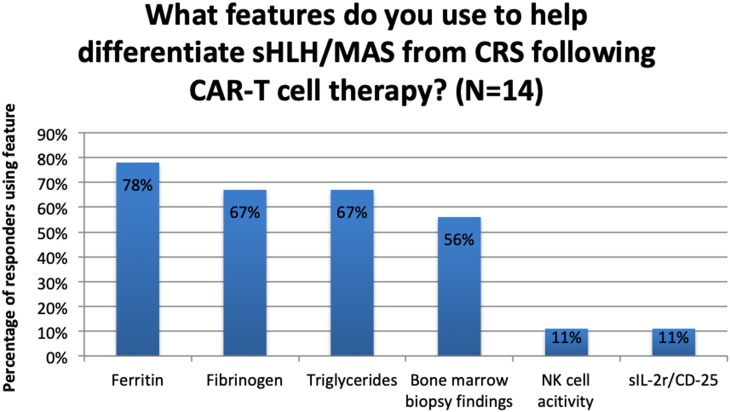
Use of laboratory features to differentiate between sHLH/MAS and CRS following CAR-T cell therapy.

Regarding CRS, 3 out of 14 centres (21%) reported that they did not use any clinical or laboratory features to help them differentiate sHLH/MAS from CRS. Of the 11 centres that did, the frequency with which laboratory parameters were used is reported in Figure 2.

### Use of Serum Ferritin in Screening for sHLH/MAS Post HSCT or CAR-T Cell Therapy

**Figure 3:**
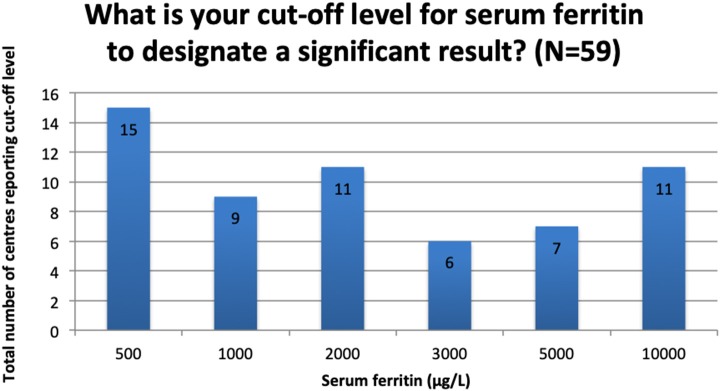
Reported cut-off levels to define a significant serum ferritin result.

**Figure 4:**
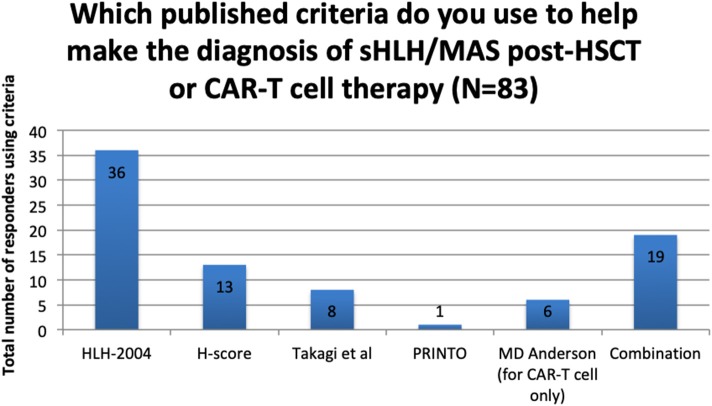
Use of published criteria to support diagnosis of sHLH/MAS post-HSCT or CAR-T cell therapy.

Though it was the most commonly reported marker of sHLH/MAS, there was great variation in what was considered a “clinically significant” serum ferritin level. The most commonly reported cut-off values are reported in Figure 3. The responses to this question were free text and a further 10 different values (not shown in Figure 4) were reported, ranging from 10 to 8,000 μg/L.

### Diagnosing sHLH/MAS Following HSCT/CAR-T Cell Therapy Using Published Criteria

Of the 104 responding centres, 21 (20%) reported using no published criteria to support the diagnosis of sHLH/MAS in these settings.

For the remaining 83 centres, the criteria in use are reported in Figure 4 and a summary of the criteria components is presented in Table 1.

### Management of sHLH/MAS

Only 20% of the 100 responding centers reported using a standard protocol for sHLH/MAS management.

Of these 20 centres using a treatment protocol, 4 used the MD Anderson recommendations (44). Other centres specifying their protocols reported using HLH-2004 (24) (*n* = 2), recommendations from La Rosee et al. (45) (*n* = 1) and the HLH-94 protocol (46) (*n* = 1) (Table 2). Whilst “international guidelines” and “HLH international society guidelines” were also reported in the survey as standard protocols, the responders did not specify to which these refer, but they may refer to the HLH-2004 guidelines produced by the Histiocyte society (24). No further specific protocols were reported.

**Table 2:** Use of published protocols in the management of sHLH/MAS post-HSCT or CAR-T cell therapy.

**Published protocol**	**Components of protocol**	**Centres (*N*)**
MD Anderson (post CAR-T cell) (44)	Supportive organ-specific treatment, broad-spectrum antibiotics, IV Tocilizumab or Siltuximab (anti-IL6 agents), IV corticosteroids	4
HLH-2004 (for fHLH) (24)	8 weeks initial therapy with IV dexamethasone and Etoposide. Then ciclosporin is introduced, dexamethasone continues to be pulsed and etoposide continued whilst awaiting a donor for BMT	2
La Rosee et al. (45)	Use of corticosteroids +/- IVIG in most cases with addition of etoposide (if malignancy-triggered), ciclosporin & anakinra (if autoimmune-related) or anti-IL-6 (if CAR-T cell related)	1
HLH-94 (for fHLH)	8 weeks initial therapy with IV dexamethasone and Etoposide before proceeding to definitive treatment with BMT	1

When asked which agents are used to treat sHLH/MAS there were 16 different responses from 97 centres. The most frequently reported combinations were corticosteroids +chemotherapy (25%), corticosteroids + monoclonal antibodies + chemotherapy (15%), corticosteroids + chemotherapy + cytokine blockade (13%), corticosteroids + cytokine blockade (12%), and corticosteroids alone (10%). In terms of specific agents reported as being used in the management of sHLH/MAS, the most common were etoposide (*n* = 17), rituximab (*n* = 8), and tocilizumab (*n* = 7). A range of other agents were reported including Cytosorb®, ruxolitinib, CSA, IVIG, anakinra, ATG, alemtuzumab, methotrexate, vincristine, baricitinib, and siltuximab.

## Discussion

We surveyed the EBMT community to assess current awareness and clinical diagnostics and management of this serious and frequently life-threatening complication of HSCT/CAR-T cell therapy. Estimates of incidence or prevalence of sHLH/MAS post-HSCT currently rely on post-hoc case reporting in a context of no agreed or validated diagnostic or therapeutic guidelines or criteria in the EBMT community. The survey reported here included 114 centres from 24 countries, a wider volume and spread than the several hundred cases reported in other publications, mainly from single centres (2, 8, 27).

We report an estimated rate following allogeneic HSCT of 1.09% and much lower estimate of 0.15% following autologous HSCT. This is slightly higher than an EBMT study including 15 centres from 2005 to 2009, which identified sHLH/MAS in 0.3% of patients (5/1,423) undergoing allogeneic HSCT (47). The estimated rate from our survey and the EBMT study are lower than incidence reports in other studies, at ~3–4% (2, 6, 8). Whilst estimated rates and formal measures of prevalence and incidence cannot be directly compared, the differences may suggest that in centres where prospective study is performed, more cases are being identified, suggesting under-recognition in general HSCT practice. These other studies used specific criteria to diagnose sHLH/MAS, whilst our survey sought to understand the heterogeneity of sHLH/MAS approaches and therefore did not limit diagnosis to such specific criteria.

We report a rate of sHLH/MAS following CAR-T cell therapy of 3.48%. Previously, sHLH/MAS has been reported in ~1% of patients undergoing CAR-T cell therapy in a single centre (44). To our knowledge this is the first formal report of rates across multiple centres. As sHLH/MAS is considered a severe manifestation of CRS, our higher diagnostic rate may reflect evolving experience of CAR-T cell therapy and greater awareness of associated current recommendations as to the detection and management of the CRS-sHLH/MAS spectrum.

Seventy per cent of centres reported using no standard screening protocols to identify sHLH/MAS post-HSCT/CAR-T cell therapy. Of those reporting their screening markers (with or without the use of a standard protocol), the most common marker was serum ferritin (71%) though this was often used in combination with fibrinogen, triglycerides, bone marrow analysis and less commonly sIL-2R or NK cell function. These, in combination, are all components of existing scores, such as the H-score and HLH-2004 criteria (24, 25). Again, this highlights a heterogeneous approach to screening amongst centres, using markers validated in other patient groups. Until robust study into reliable markers of sHLH/MAS in the post-HSCT/CAR-T cell setting is undertaken, we expect continuing diversity in approaches used throughout EBMT centres.

A clear theme of this survey was the use of serum ferritin as a screening marker and as part of diagnostic criteria. There was significant variation in what was regarded as a “significant” ferritin result. The median cut-off value deemed significant was 3,000 μg/L (IQR 1,000–10,000 μg/L). Interestingly, this median is similar to the optimum cut-off for HLH recommended by Basu et al. (48) of 3,120 μg/L (albeit in paediatric patients and not in the post-HSCT setting). As already discussed, what constitutes significant hyperferritinaemia in the post-HSCT setting is as yet undefined and further study is needed to define appropriate cut-off ranges to inform novel screening and diagnostic criteria.

Following CAR-T cell therapy specifically, serum ferritin > 10,000 μg/L is observed in patients with all grade of CRS, not just in those with the higher grades (49). Of the 9 centres that reported using specific clinical or laboratory features to make this differentiation, 7 (78%) reported the use of serum ferritin to make the distinction between CRS and SHLH/MAS. There were no direct reports of using the MD Anderson criteria suggested by Neelapu et al. (44).

In terms of diagnosis of sHLH/MAS the responders again showed a heterogeneous approach. The most frequently cited criteria were HLH-2004 (24) and the H-score (25), neither of which are validated in the HSCT setting. The only specific diagnostic criteria in the HSCT setting was produced by Takagi et al. (27), and 8 centres reported using it to aid in diagnosis. This was only studied in patients undergoing umbilical cord transplantation and has not been validated in larger, more generalised HSCT studies. The work of Abdelkefi et al. used an adapted criteria for identifying sHLH/MAS post-HSCT, incorporating bilineage cytopenia, fever, bone marrow findings and a serum ferritin > 1 000 μg/L (2, 50). With no consensus on appropriate diagnostic criteria, there is considerable variation in the definition of sHLH/MAS post-HSCT, which makes further study into this condition problematic.

In terms of management of sHLH/MAS, a majority of responders (80%) reported an absence of standard protocols, in keeping with the lack of evidence in this population. As expected, protocols which were used included the HLH-2004 protocol and seem to predominately involve use of corticosteroids ± chemotherapeutic options. Etoposide was a commonly reported agent, in keeping with the HLH-2004 recommendations, though there are concerns about using etoposide in the post-HSCT setting (24, 43). Only 35% of centres reported using cytokine blockade (in different combinations with other therapeutic classes), which has revolutionised the management of sHLH/MAS in other settings, though its benefit has not been studied in the post-HSCT setting. IVIG use was reported and has features in recent recommendations for managing sHLH/MAS in any setting (1). Ruxolitinib, a janus kinase inhibitor, use was also reported and has shown varied response in multiple case reports, including patients with EBV- and non-EBV driven HLH, but promising results in a recent pilot studies including 40 patients sHLH/MAS (51–55). Again, there is no evidence of its efficacy in the post-HSCT setting but this is an agent to consider in the future. As most CAR-T cell therapies will have been performed in clinical trials, with more rigorous monitoring and with clear management advice around CRS and sHLH/MAS than general HSCT practice, we were not surprised to find frequent use of the MD Anderson criteria in our survey (44).

This survey had several limitations. We surveyed the EBMT community with a 24.1% response rate (114/472). Therefore we have not collected data from a majority of EBMT centres, which limits the robustness of our epidemiological estimates. Furthermore, as our denominator, we took the total number of HSCT performed in a centre over the 3-year period, but did not specify if these were all “first-time” transplants. There may have been patients included multiple times in the denominator if they underwent repeated HSCT, which this study was not designed to identify. All surveys are prone to responder bias and we are aware this survey may have been preferentially responded to by groups already recognising sHLH/MAS in their post-HSCT cohorts and may not truly represent the community as a whole. However as 69/114 responding centres reported 0 cases managed we believe this bias is reasonably mitigated.

This retrospective analysis relied on the EBMT PI recalling cases of sHLH/MAS managed in the post-HSCT/CAR-T cell therapy setting over a 3-year period (2016–2018). The time period of inclusion was restricted to 2016–2018 and we consider all cases declared during this interval and all transplant activity of responding centres over the same time period. A case diagnosed in 2016 could have been related to a transplant performed before 2016 and some cases related to transplants between 2016 and 2018 could only be diagnosed after 2018. The design of the survey didn't allow such discrimination. Furthermore, prospective, rather than retrospective studies which are prone to bias, are favoured in providing accurate incidence estimates and we should consider this in future work (56). We asked PIs to report on the number of cases they had diagnosed but did not scrutinise how this diagnosis was made, in comparison to previous smaller-centre reports, which have used specific criteria (2, 8, 27). Cases may simply have been forgotten by the clinician or incorrectly diagnosed in the past or not recognised, which is a limitation of this work, however, with this being a rare and often devastating complication we hoped cases would be retained and recalled by EBMT centres. The design of this survey did not allow for review of mortality in this cohort but it has been reported up to 83% in recent case reviews (6, 7).

## Conclusion

Secondary HLH/MAS is a relatively rare and serious complication of HSCT and CAR-T cell therapy, which is heterogeneously defined and managed in the sampled EBMT community. Dedicated study is warranted to design and evaluate protocols for screening, diagnosis, and management.

## Data Availability Statement

The datasets generated for this study are available on request to the corresponding author.

## Author Contributions

JS, RDS, and RT conceptualised this paper. TA, KK, RG, MR, MS, RS, ZP, HS, JD, CK, OP, and GB were involved in design and phrasing of the survey. MB was responsible for coordinating the survey through the EBMT office. ML was responsible for design of methodology and epidemiological statistics. RDS drafted the initial manuscript and revised it according to feedback from all authors, who were involved in critical revisions and provided important intellectual content.

## Acknowledgements

We acknowledge the 114EBMT centers who responded to this survey and the EBMT data office in Paris, France for their assistance in obtaining and analysing the data. We acknowledge the EBMT ADWP and TCWP for support of this work.

### Conflict of Interest

JS declares speaker fees from Sanofi, Gilead, Jazz, Mallinckrodt, and Janssen and is a trial IDMC member for Kiadis. RDS declares conference attendance as a sponsored delegate of Lilly. The remaining authors declare that the research was conducted in the absence of any commercial or financial relationships that could be construed as a potential conflict of interest.

## Abbreviations

sHLH/MAS: Secondary haemophagocytic lymphohistiocytosis/macrophage activation syndrome
HSCT: haematopoetic stem cell transplantation
CRS: cytokine release syndrome.
