# Supplementary material for: Diagnosis and Management of Secondary HLH/MAS Following HSCT and CAR-T Cell Therapy in Adults; A Review of the Literature and a Survey of Practice Within EBMT Centres on Behalf of the Autoimmune Diseases Working Party (ADWP) and Transplant Complications Working Party (TCWP)
Source: Front Immunol. 2020 Mar 31;11:524. doi: 10.3389/fimmu.2020.00524 (PMC7137396; doi:10.3389/fimmu.2020.00524)
Supplement: Supplementary file 1 [file Data_Sheet_1.pdf]

European Society
for Blood and Marrow
Transplantation

Welcome to the EBMT survey of sHLH/MAS post-HSCT or CAR-T therapy. Please complete the following questions regarding adult patients (aged 18 and over) managed in your centre in 2016, 2017 and 2018 inclusive.

Question (Q) 1 to 4 : identification of centres

Q5. How many cases of sHLH/MAS following **autologous** HSCT are you aware of in your centre between 2016-2018 inclusive (we will find the total number of transplantations for your CIC from the EBMT registry)

0	1	2	3	4	5	6	7	8	9	10
○	○	○	○	◉	○	○	○	○	○	○

Q6. How many cases of sHLH/MAS following **allogeneic** HSCT are you aware of in your centre between 2016-2018 inclusive (we will find the total number of transplantations for your CIC from the EBMT registry)

0	1	2	3	4	5	6	7	8	9	10
○	○	○	○	○	◉	○	○	○	○	○

Q7. Have you performed CAR-T therapy in your centre?

◉ No ○ Yes

If yes

Q7a. Please state the total number of patients treated with CAR-T therapy (on or off trial) in your centre between 2016-2018 inclusive

Q7b. How many cases of sHLH/MAS, following CAR-T therapy, are you aware of in your centre between 2016-2018 inclusive?

0	1	2	3	4	5	6	7	8	9	10
○	○	○	○	○	○	○	○	○	○	○

Q8. In your programme, do you use published criteria for sHLH/MAS to support diagnosis post HSCT and CAR-T cell therapy?

(•) No ( ) Yes

if yes. Which published criteria are you using ? (see below for details of criteria) (Tick all that apply)

- [ ] HLH-2004 criteria (Henter et al, 2007)
- [ ] H-score (Fardet et al, 2014)
- [ ] HLH/MAS post-HSCT criteria (Takagi et al, 2009)
- [ ] PRINTO (Ravelli et al, 2016)
- [ ] MD Anderson criteria (FOR CAR-T only) (Neelapu et al, 2018)
- [ ] Other, specify ______

Q9. In your programme, do you have an approved protocol or SOP to pre-emptively screen for sHLH/MAS in patients post-HSCT or CAR-T cell therapy?

(•) No ( ) Yes

Q9.a. **Autologous** HSCT: do you have an approved protocol or SOP to pre-emptively screen for sHLH/MAS in patients post-autologous HSCT?

- ( ) No
- ( ) Yes, when the clinical picture is suspicious for sHLH/MAS
- ( ) Yes, routinely

Q9.a (i). Frequency of screening after **autologous** HSCT

- ( ) Daily
- ( ) Weekly
- ( ) Fortnightly (every 2 weeks)
- ( ) Monthly
- ( ) Other ______

Q9.a (ii). For how long do you screen these patients after **autologous** HSCT?

- 3 months
- 6 months
- 12 months
- Other

Q9.b Allogeneic HSCT: do you have an approved protocol or SOP to pre-emptively screen for sHLH/MAS in patients post-allogeneic HSCT?

- No
- Yes, when the clinical picture is suspicious for sHLH/MAS
- Yes, routinely

Q9.b (i). Frequency of screening after **allogeneic** HSCT

- Daily
- Weekly
- Fortnightly (every 2 weeks)
- Monthly
- Other

Q9.b (ii). For how long do you screen these patients after allogeneic HSCT?

- 3 months
- 6 months
- 12 months
- Other

Q9.c CAR-T : do you have an approved protocol or SOP to pre-emptively screen for sHLH/MAS in patients post-CAR-T therapy?

- No
- Yes, when the clinical picture is suspicious for sHLH/MAS
- Yes, routinely

Q9.c (i). Frequency of screening after CAR-T therapy

- Daily
- Weekly
- Fortnightly (every 2 weeks)

- Monthly
- Other [ ]

Q9.c (ii) For how long do you screen these patients after CAR-T therapy?

- 3 months
- 6 months
- 12 months
- Other [ ]

Q10. Do any clinical or laboratory features help you differentiate between sHLH/MAS and severe cytokine-release syndrome (CRS) following CAR-T therapy?

[ ]

Q11. If you do perform screening, what are your screening markers? (Tick all that apply)

- [ ] Serum ferritin
- [ ] Soluble CD25/sIL-2r
- [ ] NK cell activity
- [ ] Other [ ]

Q11.a Serum ferritin: please indicate what measurement is considered a significant result

[ ]

Q11.b Soluble CD25/sIL-2r : please indicate what measurement is considered a significant result

[ ]

Q11.c NK cell activity : please indicate what measurement is considered a significant result

[ ]

Q11.d.For other drugs : please indicate what measurement is considered a significant result for each drug

[ ]

Q12. .If you use ferritin, what is your cut off to help determine likelihood of sHLH/MAS (free text)?

[ ]

Q13. Do you have an approved protocol or SOP to treat sHLH/MAS in patients post-HSCT and CAR-T cell therapy?

No   Yes

Q13.a. If yes, describe its basis e.g. does it follow international recommendations for management of sHLH/MAS? Specify which.

Q14. In your programme, which agents would/do you use to treat sHLH/MAS? (tick all that apply)

- Corticosteroids
- Monoclonal antibody therapy (e.g. rituximab, alemtuzumab, emapalumab other please specify)
- Chemotherapy (e.g. etoposide, methotrexate, other please specify)
- JAK inhibitors (e.g. baricitinib, other please specify)
- Cytokine blockade (e.g. tocilizumab, siltuximab, anakinra)
- Other supportive care, please specify

Q14.a Please specify which one(s)

Q14.b If you use corticosteroids

- IV pulses only
- IV followed by oral
- Oral only
- Other

Q15. If you have indicated that you have managed cases of sHLH/MAS following HSCT or CAR-T therapy, are you happy to be contacted in the future about a retrospective study of these patients?

- No
- Yes
- Other
